# Supplementary material for: Incidence and risk factors of SARS-CoV-2 breakthrough infection in the early Omicron variant era among vaccinated and boosted individuals in Chicago
Source: PLoS One. 2024 Aug 5;19(8):e0302338. doi: 10.1371/journal.pone.0302338 (PMC11299831; doi:10.1371/journal.pone.0302338)
Supplement: Attachment — Submitted filename: Response to Reviewers.docx [file pone.0302338.s004.docx]

**Response to Reviewers: Editor/Reviewer Points (***italics***) and** Rebuttal

For each rebuttal, references to Page and Line numbers are for the ‘Revised Manuscript with Tracked Changes’ document.

**Reviewer 1**

1. *In table 1, the percentages should be calculated across the rows among the groups to accurately reflect the breakthrough infections among the populations. For instance, in the "Boosted 2+"(total is 1,025), the no breakthrough and breakthrough infections are 982 (0.8%) and 43 (0.5%) respectively. Rather, it should be 982/1025 (95.8%) and 43/1025 (4.2%). Similarly, in the "Boosted once" it will be 124,434/132,279 (94.1%) and 7,845/132,279 (5.9%) for the no breakthrough and breakthrough infections respectively. In this case you can determine if the difference is significant between Boosted 2+ and boosted once. The percentages for the "overall" column is okay.*

Response/Revision: Thank you for this comment. We agree that presenting row percentages in the stratified columns allows for direct comparison of infection rates between categories of characteristics. We have adjusted the table accordingly. We have made these changes in the Results section on page 6, line 129.

2. *The study aims to "understand the epidemiology of Omicron breakthrough infection and evaluate associations with number of comorbidities in a vaccinated and boosted population". A further analysis of individuals with comorbidities and boosters will provide additional data to answer the study's aim.*

Response/Revision: We would like to clarify for this reviewer why we did not evaluate associations between the boosters received and breakthrough SARS-CoV-2 infection. First, and foremost, as presented in Table 1 of our manuscript, 99% of individuals were boosted with mRNA SARS-CoV-2 vaccines (Moderna or Pfizer). At this point in the COVID-19 pandemic, these two mRNA boosters displayed similar effectiveness and were administered interchangeably. Also, we had only 59 (0.7%) breakthrough infections occurring among individuals who received non-mRNA boosters. Thus, we did not believe that we had a compelling enough scientific or statistical rationale to conduct analyses on type of boosters received or to include this factor in multivariable Cox models. As stated above, our aim was to understand the epidemiology and risk factors for breakthrough Omicron infection (focusing on burden of comorbidities) among a vaccinated and boosted population.

3. *In the results section, in table 2, the authors divided the study period into 2 that is period 1 and period 2. Though the reason is given for the separated study periods, this is not mentioned in the methods. The authors should let the reader be aware of the study periods considered in the method.*

*Response/Revision:* Thank you for this comment.  We agree that the methods that led to dividing the study period should be clarified in the Methods section. We added text to Methods page 6 (lines 115 – 116) that clarifies the approach we used to make this determination.

4. *In the results section, the authors mentioned a 5.9% SARS-CoV-2 infection while in the discussion section, they mentioned 5.1% cumulative incidence. This should be checked and rectified.*

*Response/Revision:* Thank you for pointing out this error. The statistic referenced in the Discussion has been corrected to 5.9%. See Discussion on page 11 (line 177).

5. *If the breakthrough infection is calculated across the groups, I think the infection breakthrough among individuals with multiple comorbidities will be approximately 6% and not the percentages indicated. This will address statements in the discussion section such as "We observed a higher incidence of breakthrough infections among individuals with multiple comorbidities and those who had a longer time since vaccine booster.*

Response/Revision: Thank you for pointing this out. These unadjusted cumulative incidences are in fact similar; however, we did observe a significant increase in adjusted hazard of breakthrough infection in multivariable Cox modeling after controlling confounders of age, sex, race, ethnicity, and time from boosting. The discussion has been adjusted to reflect the correct findings. See Discussion on page 11 (Lines 177 – 180).

**Reviewer #2**

1. *Authors could consider identifying person/place/time in the title.*

Response/Revision: We agree that our title would be more informative if it clearly identifies person/place/time. Thus, we have changed the title to “Incidence and risk factors of SARS-CoV-2 breakthrough infection in the early Omicron variant era among vaccinated and boosted individuals in Chicago” on page 1.

2. *All observations were not particularly linked to the biology of the Omicron variant.*

Response/Revision: We would like to clarify for this reviewer that we did not have individual level SARS-CoV-2 sequencing information on breakthrough infections, and thus, were unable to assess whether they were definitively due to Omicron variant. However, from surveillance SARS-CoV-2 sequencing studies in Chicago, we know that nearly 100% of circulating SARS-CoV-2 viruses after January 1, 2022 were due to the Omicron variant, thus we can safely assume that all of the patients studied in this analysis had Omicron and sub-variants. Throughout the manuscript we were careful to use the phrase “Omicron variant era” since we did not have individual sequencing information. We also changed the title on page 1 to now state “…early Omicron variant era…”

3. *Selected population is aged (44% 60–79-year-olds), could this have biased the findings?*

Response/Revision: We agree that throughout the COVID-19 pandemic, age has been an important factor and driving force behind risk for severe disease and behavioral risk modification. To account for this, we adjusted for age as a confounder in our Cox models and these adjusted hazard ratios interpretated in the Discussion section suggest that even after controlling for age, break through infections were associated with number of co-morbidities and time since vaccine boosting. Thus, we don’t believe that the distribution of age across our cohort influences the robustness or direction of our findings.

4. *On line 115, for population characteristics, you state 84% white, 95% non-hispanic/Latina. This is not very clear. Is it that the population was made up of 95% non-hispanic/Latina of which 84% are white?*

Response/Revision: Thank you for bringing this up to our attention. We reworded the sentence for clarity. Our total population was made up of 84% white race. In terms of the ethnicity of the total population, the majority (95%) identified as non-Hispanic. All the percentages described are of the total population.

See Results section on page 6 (Line 124-126) “In total, there were 133,191 patients in the cohort with a median (IQR) age of 61 years (47, 72) 63% female sex, 84% white race, and 77% with any comorbid condition. Of the total population, 5.3% identified as Hispanic/Latino”

**Reviewer #3**

1. *Line 37(method and findings): Please clarify in the abstract the 2 phases of the study and why the study period was divided into 2 periods.*

Response/Revision: Thank you for the comment. The study was divided into two periods because after assessing cumulative incidence curves for each covariate the proportional hazards assumption was violated. Thus, for covariates that violated this assumption, graphical assessment of incidence rate trends was performed to evaluate for significant changes over time. This required introducing an interaction term and calculation of separate hazard ratios for two time periods.

We have specified the two study periods in the abstract and commented on why this was done. See Abstract, page 2 (Line 33-36): “We performed multivariable analyses stratified by calendar time (Period 1: January 1 – June 30, 2023; Period 2: July 1 – December 31, 2023) using Cox modeling to determine hazard of SARS-CoV-2 due to violations in proportional hazards assumption"

2. *Line 43 (conclusion): You concluded that behavioral factors play an important role in breakthrough infection.” Age related behavioral factors play an important role in breakthrough infection with the highest incidence among young adults.”. However, behavioral factors were not studied in this cohort.*

Response/Revision: Thank you. It is correct that we did not directly study behavioral factors. We adjusted the wording to be more speculative as based on available literature it may be a driving factor of the observed age differences in breakthrough infections, but not directly studied here (Page 2, line 44-46.)

3. *Our study aimed and was able to determine the incidence and risk factors associated with SARS-CoV-2…etc.” Result was included in the introduction. Delete “and was able”*

*Response/Revision:* Thank you. We have deleted the result from the introduction. See introduction on page 4, line 74.

4. *Line 121-122: “The infections occurred in a median (IQR) of 196 (115, 260) days after a booster dose of vaccine. Could you justify the use of the median(IQR) here, and how did you get the 196-days figure???*

Response/Revision l: We chose to use median (IQR) as this statistic is only meant to be descriptive and we believe it is clearer for the reader than mean (SD). Additionally, its interpretation doesn’t rely on normality.

The calculation for the 196-day figure was simply adding 1) days from booster to study start, and 2) days from study start to infection. We added this to the Statistical Analysis section:

“For those with breakthrough infection, we calculated the median (IQR) time to infection from booster by summing the number of days from booster to study start and from study start to infection date. “

To clarify that this data was collected, we added the median (IQR) for the Days since booster variable in Table 1 to supplement the categories.

See Methods page 5 (line 106-108) and Results page 9 (line 130).

5. *This data could be presented better visually. I suggest a graphic representation of this data*

Response/Revision: Thank you for this feedback. We agree that visual representation may be a better representation of this data. Multivariable analysis data (Fig. 1) is now presented in a forest plot to more effectively display and summarize the effect on breakthrough infection hazard of the various variables in each study period. This new figure has been uploaded in the resubmission. See figure caption on page 9 line 148.

6. *There are a few typo-grammatical errors throughout the manuscript including the references. I highlighted some of them on the pdf file. Please address these errors. Avoid using 1st person pronouns.*

Response/Revision: Thank you. Grammatical errors were addressed and sentences using first person pronouns were rephrased throughout the manuscript.
