# Supplementary material for: Incidence and risk factors of SARS-CoV-2 breakthrough infection in the early Omicron variant era among vaccinated and boosted individuals in Chicago
Source: PLoS One. 2024 Aug 5;19(8):e0302338. doi: 10.1371/journal.pone.0302338 (PMC11299831; doi:10.1371/journal.pone.0302338)
Supplement: Peer review history

# Peer review: Decision Letter 0

**DOI:** 10.1371/journal.pone.0302338.r001

**Authors:** Olatunji Matthew Kolawole

27 May 2024

PONE-D-24-12478Incidence and risk factors of omicron variant SARS-CoV-2 breakthrough infection among vaccinated and boosted individuals.PLOS ONE

Dear Dr. Achenbach,

Thank you for submitting your manuscript to PLOS ONE. After careful consideration, we feel that it has merit but does not fully meet PLOS ONE’s publication criteria as it currently stands. Therefore, we invite you to submit a revised version of the manuscript that addresses the points raised during the review process.

**ACADEMIC EDITOR: Kindly respond to all comments made by reviewers and make appropriate review of the manuscript where necessary.**

Please submit your revised manuscript by Jul 11 2024 11:59PM. If you will need more time than this to complete your revisions, please reply to this message or contact the journal office at plosone@plos.org. When you're ready to submit your revision, log on to https://www.editorialmanager.com/pone/ and select the 'Submissions Needing Revision' folder to locate your manuscript file.

Please include the following items when submitting your revised manuscript:

- A rebuttal letter that responds to each point raised by the academic editor and reviewer(s). You should upload this letter as a separate file labeled 'Response to Reviewers'.
- A marked-up copy of your manuscript that highlights changes made to the original version. You should upload this as a separate file labeled 'Revised Manuscript with Track Changes'.
- An unmarked version of your revised paper without tracked changes. You should upload this as a separate file labeled 'Manuscript'.

If you would like to make changes to your financial disclosure, please include your updated statement in your cover letter. Guidelines for resubmitting your figure files are available below the reviewer comments at the end of this letter.

If applicable, we recommend that you deposit your laboratory protocols in protocols.io to enhance the reproducibility of your results. Protocols.io assigns your protocol its own identifier (DOI) so that it can be cited independently in the future. For instructions see: https://journals.plos.org/plosone/s/submission-guidelines#loc-laboratory-protocols. Additionally, PLOS ONE offers an option for publishing peer-reviewed Lab Protocol articles, which describe protocols hosted on protocols.io. Read more information on sharing protocols at https://plos.org/protocols?utm_medium=editorial-email&utm_source=authorletters&utm_campaign=protocols.

We look forward to receiving your revised manuscript.

Kind regards,

Olatunji Matthew Kolawole, Ph.D.

Academic Editor

PLOS ONE

Journal requirements:

When submitting your revision, we need you to address these additional requirements.

1. Please ensure that your manuscript meets PLOS ONE's style requirements, including those for file naming. The PLOS ONE style templates can be found at

https://journals.plos.org/plosone/s/file?id=wjVg/PLOSOne_formatting_sample_main_body.pdf and

https://journals.plos.org/plosone/s/file?id=ba62/PLOSOne_formatting_sample_title_authors_affiliations.pdf

2. We note that your Data Availability Statement is currently as follows: [All relevant data are within the manuscript and its Supporting Information files.]

Please confirm at this time whether or not your submission contains all raw data required to replicate the results of your study. Authors must share the “minimal data set” for their submission. PLOS defines the minimal data set to consist of the data required to replicate all study findings reported in the article, as well as related metadata and methods (https://journals.plos.org/plosone/s/data-availability#loc-minimal-data-set-definition).

For example, authors should submit the following data:

- The values behind the means, standard deviations and other measures reported;

- The values used to build graphs;

- The points extracted from images for analysis.

Authors do not need to submit their entire data set if only a portion of the data was used in the reported study.

If your submission does not contain these data, please either upload them as Supporting Information files or deposit them to a stable, public repository and provide us with the relevant URLs, DOIs, or accession numbers. For a list of recommended repositories, please see https://journals.plos.org/plosone/s/recommended-repositories.

If there are ethical or legal restrictions on sharing a de-identified data set, please explain them in detail (e.g., data contain potentially sensitive information, data are owned by a third-party organization, etc.) and who has imposed them (e.g., an ethics committee). Please also provide contact information for a data access committee, ethics committee, or other institutional body to which data requests may be sent. If data are owned by a third party, please indicate how others may request data access.

3. Please review your reference list to ensure that it is complete and correct. If you have cited papers that have been retracted, please include the rationale for doing so in the manuscript text, or remove these references and replace them with relevant current references. Any changes to the reference list should be mentioned in the rebuttal letter that accompanies your revised manuscript. If you need to cite a retracted article, indicate the article’s retracted status in the References list and also include a citation and full reference for the retraction notice.

[Note: HTML markup is below. Please do not edit.]

Reviewers' comments:

Reviewer's Responses to Questions

**Comments to the Author**

1. Is the manuscript technically sound, and do the data support the conclusions?

The manuscript must describe a technically sound piece of scientific research with data that supports the conclusions. Experiments must have been conducted rigorously, with appropriate controls, replication, and sample sizes. The conclusions must be drawn appropriately based on the data presented. 

Reviewer #1: Partly

Reviewer #2: Yes

Reviewer #3: Partly

**********

2. Has the statistical analysis been performed appropriately and rigorously? 

Reviewer #1: I Don't Know

Reviewer #2: Yes

Reviewer #3: No

**********

3. Have the authors made all data underlying the findings in their manuscript fully available?

The PLOS Data policy requires authors to make all data underlying the findings described in their manuscript fully available without restriction, with rare exception (please refer to the Data Availability Statement in the manuscript PDF file). The data should be provided as part of the manuscript or its supporting information, or deposited to a public repository. For example, in addition to summary statistics, the data points behind means, medians and variance measures should be available. If there are restrictions on publicly sharing data—e.g. participant privacy or use of data from a third party—those must be specified.

Reviewer #1: Yes

Reviewer #2: Yes

Reviewer #3: Yes

**********

4. Is the manuscript presented in an intelligible fashion and written in standard English?

PLOS ONE does not copyedit accepted manuscripts, so the language in submitted articles must be clear, correct, and unambiguous. Any typographical or grammatical errors should be corrected at revision, so please note any specific errors here.

Reviewer #1: Yes

Reviewer #2: Yes

Reviewer #3: Yes

**********

5. Review Comments to the Author

Please use the space provided to explain your answers to the questions above. You may also include additional comments for the author, including concerns about dual publication, research ethics, or publication ethics. (Please upload your review as an attachment if it exceeds 20,000 characters)

Reviewer #1: Major

1. In table 1, the percentages should be calculated across the rows among the groups to accurately reflection of the breakthrough infections among the populations. For instance, in the "Boosted 2+"(total is 1,025), the no breakthrough and breakthrough infections are 982 (0.8%) and 43 (0.5%) respectively. Rather, it should be be 982/1025 (95.8%) and the 43/1025 (4.2%). Similarly, in the "Boosted once" it will be 124,434/132,279 (94.1%) and 7,845/132,279 (5.9%) for the no breakthrough and breakthrough infections respectively. In this case you can determine if the difference is significant between Boosted 2+ and Boosted once. The percentages for the "overall" column is okay.

2. The study aims to "understand the epidemiology of Omicron breakthrough infection and evaluate associations with number of comorbidities in a vaccinated and boosted population". A further analysis of the individuals with comorbidities and the boosters received will provide additional data to answer the aim of the study.

Minor

1. In the results section, in table 2, the authors divided the study period into 2 that is period 1 and period 2. Though the reason is given for the separated study periods, this is not mentioned in the methods. The authors should let the reader be aware of the study periods considered in the method.

2. In the results section, the authors mentioned a 5.9% SARS-CoV-2 infection while in the discussion section, they mentioned 5.1% cumulative incidence. This should be checked and rectified.

3. If the breakthrough infection is calculated across the groups, I think the infection breakthorugh among individuals with multiple comorbidities will be approximately 6% and not the percentages indicated. This will address statements in the discussion section such as "We observed a higher incidence of breakthrough infections among individuals with multiple comorbidities and those who had a longer time since vaccine booster".

Reviewer #2: This study aimed to investigate the incidence and risk factors that contribute to breakthrough SARS-CoV-2 infections (omicron) among vaccinated and boosted individuals. This was an observational study, describing what was observed during this period.

1. Authors could consider identifying person/place/time in the title.

2. All observations were not particularly linked to the biology of the omicron variant

3. Selected population is aged (44% 60-79 year olds), could this have biased the findings?

4. On line 115, for population characteristics, you state 84% white, 95% non-hispanic/Latina. This is not very clear. Is it that the population was made up of 95% non-hispanic/Latina of which 84% are white?

Reviewer #3: Dear Authors,

Thank you for submitting your research for publication. This is a well-structured manuscript. However, there are areas for improvement.

Please address the following points:

Abstract:

Line 37(method and findings): Please clarify in the abstract the 2 phases of the study and why the study period was divided into 2 periods.

Line 43 (conclusion): You concluded that behavioral factors play an important role in breakthrough infection. ” Age related behavioral factors play an important role in breakthrough infection with the highest incidence among young adults.”. However, behavioral factors were not studied in this cohort.

Introduction:

Line 70: “Our study aimed and was able to determine the incidence and risk factors associated with SARS-CoV-2…etc.” Result was included in the introduction. Delete “and was able”

Result:

Table 1: Would you please clarify where the median IQR was used in Table 1?

Line 121-122: “ The infections occurred in a median (IQR) of 196 (115, 260) days after a booster dose of vaccine. Could you justify the use of the median(IQR) here, and how did you get the 196-days figure???

Table 2: This data could be presented better visually. I suggest a graphic representation of this data

General points:

There are a few typo-grammatical errors throughout the manuscript including the references. I highlighted some of them on the pdf file. Please address these errors.

Avoid using 1st person pronouns.

Best wishes

Kind regards

**********

6. PLOS authors have the option to publish the peer review history of their article (what does this mean?). If published, this will include your full peer review and any attached files.

If you choose “no”, your identity will remain anonymous but your review may still be made public.

**Do you want your identity to be public for this peer review?** For information about this choice, including consent withdrawal, please see our Privacy Policy.

Reviewer #1: No

Reviewer #2: No

Reviewer #3: No

**********

[NOTE: If reviewer comments were submitted as an attachment file, they will be attached to this email and accessible via the submission site. Please log into your account, locate the manuscript record, and check for the action link "View Attachments". If this link does not appear, there are no attachment files.]

While revising your submission, please upload your figure files to the Preflight Analysis and Conversion Engine (PACE) digital diagnostic tool, https://pacev2.apexcovantage.com/. PACE helps ensure that figures meet PLOS requirements. To use PACE, you must first register as a user. Registration is free. Then, login and navigate to the UPLOAD tab, where you will find detailed instructions on how to use the tool. If you encounter any issues or have any questions when using PACE, please email PLOS at figures@plos.org. Please note that Supporting Information files do not need this step.

Attachment Submitted filename: PONE-D-24-12478_reviewer.pdf

# Author comment: Author response to Decision Letter 0

**DOI:** 10.1371/journal.pone.0302338.r002

11 Jul 2024

For each rebuttal, references to Page and Line numbers are for the ‘Revised Manuscript with Tracked Changes’ document.

Reviewer 1 

1. In table 1, the percentages should be calculated across the rows among the groups to accurately reflect the breakthrough infections among the populations. For instance, in the "Boosted 2+"(total is 1,025), the no breakthrough and breakthrough infections are 982 (0.8%) and 43 (0.5%) respectively. Rather, it should be 982/1025 (95.8%) and 43/1025 (4.2%). Similarly, in the "Boosted once" it will be 124,434/132,279 (94.1%) and 7,845/132,279 (5.9%) for the no breakthrough and breakthrough infections respectively. In this case you can determine if the difference is significant between Boosted 2+ and boosted once. The percentages for the "overall" column is okay.

Response/Revision: Thank you for this comment. We agree that presenting row percentages in the stratified columns allows for direct comparison of infection rates between categories of characteristics. We have adjusted the table accordingly. We have made these changes in the Results section on page 6, line 129. 

2. The study aims to "understand the epidemiology of Omicron breakthrough infection and evaluate associations with number of comorbidities in a vaccinated and boosted population". A further analysis of individuals with comorbidities and boosters will provide additional data to answer the study's aim. 

Response/Revision: We would like to clarify for this reviewer why we did not evaluate associations between the boosters received and breakthrough SARS-CoV-2 infection. First, and foremost, as presented in Table 1 of our manuscript, 99% of individuals were boosted with mRNA SARS-CoV-2 vaccines (Moderna or Pfizer). At this point in the COVID-19 pandemic, these two mRNA boosters displayed similar effectiveness and were administered interchangeably. Also, we had only 59 (0.7%) breakthrough infections occurring among individuals who received non-mRNA boosters. Thus, we did not believe that we had a compelling enough scientific or statistical rationale to conduct analyses on type of boosters received or to include this factor in multivariable Cox models. As stated above, our aim was to understand the epidemiology and risk factors for breakthrough Omicron infection (focusing on burden of comorbidities) among a vaccinated and boosted population. 

3. In the results section, in table 2, the authors divided the study period into 2 that is period 1 and period 2. Though the reason is given for the separated study periods, this is not mentioned in the methods. The authors should let the reader be aware of the study periods considered in the method.

Response/Revision: Thank you for this comment. We agree that the methods that led to dividing the study period should be clarified in the Methods section. We added text to Methods page 6 (lines 115 – 116) that clarifies the approach we used to make this determination.

4. In the results section, the authors mentioned a 5.9% SARS-CoV-2 infection while in the discussion section, they mentioned 5.1% cumulative incidence. This should be checked and rectified.

Response/Revision: Thank you for pointing out this error. The statistic referenced in the Discussion has been corrected to 5.9%. See Discussion on page 11 (line 177). 

5. If the breakthrough infection is calculated across the groups, I think the infection breakthrough among individuals with multiple comorbidities will be approximately 6% and not the percentages indicated. This will address statements in the discussion section such as "We observed a higher incidence of breakthrough infections among individuals with multiple comorbidities and those who had a longer time since vaccine booster.

Response/Revision: Thank you for pointing this out. These unadjusted cumulative incidences are in fact similar; however, we did observe a significant increase in adjusted hazard of breakthrough infection in multivariable Cox modeling after controlling confounders of age, sex, race, ethnicity, and time from boosting. The discussion has been adjusted to reflect the correct findings. See Discussion on page 11 (Lines 177 – 180). 

Reviewer #2

1. Authors could consider identifying person/place/time in the title.

Response/Revision: We agree that our title would be more informative if it clearly identifies person/place/time. Thus, we have changed the title to “Incidence and risk factors of SARS-CoV-2 breakthrough infection in the early Omicron variant era among vaccinated and boosted individuals in Chicago” on page 1.

2. All observations were not particularly linked to the biology of the Omicron variant.

Response/Revision: We would like to clarify for this reviewer that we did not have individual level SARS-CoV-2 sequencing information on breakthrough infections, and thus, were unable to assess whether they were definitively due to Omicron variant. However, from surveillance SARS-CoV-2 sequencing studies in Chicago, we know that nearly 100% of circulating SARS-CoV-2 viruses after January 1, 2022 were due to the Omicron variant, thus we can safely assume that all of the patients studied in this analysis had Omicron and sub-variants. Throughout the manuscript we were careful to use the phrase “Omicron variant era” since we did not have individual sequencing information. We also changed the title on page 1 to now state “…early Omicron variant era…” 

3. Selected population is aged (44% 60–79-year-olds), could this have biased the findings?

Response/Revision: We agree that throughout the COVID-19 pandemic, age has been an important factor and driving force behind risk for severe disease and behavioral risk modification. To account for this, we adjusted for age as a confounder in our Cox models and these adjusted hazard ratios interpretated in the Discussion section suggest that even after controlling for age, break through infections were associated with number of co-morbidities and time since vaccine boosting. Thus, we don’t believe that the distribution of age across our cohort influences the robustness or direction of our findings.

4. On line 115, for population characteristics, you state 84% white, 95% non-hispanic/Latina. This is not very clear. Is it that the population was made up of 95% non-hispanic/Latina of which 84% are white?

Response/Revision: Thank you for bringing this up to our attention. We reworded the sentence for clarity. Our total population was made up of 84% white race. In terms of the ethnicity of the total population, the majority (95%) identified as non-Hispanic. All the percentages described are of the total population. 

See Results section on page 6 (Line 124-126) “In total, there were 133,191 patients in the cohort with a median (IQR) age of 61 years (47, 72) 63% female sex, 84% white race, and 77% with any comorbid condition. Of the total population, 5.3% identified as Hispanic/Latino”

Reviewer #3

1. Line 37(method and findings): Please clarify in the abstract the 2 phases of the study and why the study period was divided into 2 periods.

Response/Revision: Thank you for the comment. The study was divided into two periods because after assessing cumulative incidence curves for each covariate the proportional hazards assumption was violated. Thus, for covariates that violated this assumption, graphical assessment of incidence rate trends was performed to evaluate for significant changes over time. This required introducing an interaction term and calculation of separate hazard ratios for two time periods. 

We have specified the two study periods in the abstract and commented on why this was done. See Abstract, page 2 (Line 33-36): “We performed multivariable analyses stratified by calendar time (Period 1: January 1 – June 30, 2023; Period 2: July 1 – December 31, 2023) using Cox modeling to determine hazard of SARS-CoV-2 due to violations in proportional hazards assumption"

2. Line 43 (conclusion): You concluded that behavioral factors play an important role in breakthrough infection.” Age related behavioral factors play an important role in breakthrough infection with the highest incidence among young adults.”. However, behavioral factors were not studied in this cohort.

Response/Revision: Thank you. It is correct that we did not directly study behavioral factors. We adjusted the wording to be more speculative as based on available literature it may be a driving factor of the observed age differences in breakthrough infections, but not directly studied here (Page 2, line 44-46.)

3. Our study aimed and was able to determine the incidence and risk factors associated with SARS-CoV-2…etc.” Result was included in the introduction. Delete “and was able”

Response/Revision: Thank you. We have deleted the result from the introduction. See introduction on page 4, line 74. 

4. Line 121-122: “The infections occurred in a median (IQR) of 196 (115, 260) days after a booster dose of vaccine. Could you justify the use of the median(IQR) here, and how did you get the 196-days figure??? 

Response/Revision l: We chose to use median (IQR) as this statistic is only meant to be descriptive and we believe it is clearer for the reader than mean (SD). Additionally, its interpretation doesn’t rely on normality. 

The calculation for the 196-day figure was simply adding 1) days from booster to study start, and 2) days from study start to infection. We added this to the Statistical Analysis section: 

“For those with breakthrough infection, we calculated the median (IQR) time to infection from booster by summing the number of days from booster to study start and from study start to infection date. “ 

To clarify that this data was collected, we added the median (IQR) for the Days since booster variable in Table 1 to supplement the categories.

See Methods page 5 (line 106-108) and Results page 9 (line 130).

5. This data could be presented better visually. I suggest a graphic representation of this data

Response/Revision: Thank you for this feedback. We agree that visual representation may be a better representation of this data. Multivariable analysis data (Fig. 1) is now presented in a forest plot to more effectively display and summarize the effect on breakthrough infection hazard of the various variables in each study period. This new figure has been uploaded in the resubmission. See figure caption on page 9 line 148. 

6. There are a few typo-grammatical errors throughout the manuscript including the references. I highlighted some of them on the pdf file. Please address these errors. Avoid using 1st person pronouns.

Response/Revision: Thank you. Grammatical errors were addressed and sentences using first person pronouns were rephrased throughout the manuscript.

Attachment Submitted filename: Response to Reviewers.docx

# Editor report: Decision Letter 1

**DOI:** 10.1371/journal.pone.0302338.r003

**Authors:** Olatunji Matthew Kolawole

23 Jul 2024

Incidence and risk factors of SARS-CoV-2 breakthrough infection in the early Omicron variant era among vaccinated and boosted individuals in Chicago

PONE-D-24-12478R1

Dear Dr. Achenbach,

We’re pleased to inform you that your manuscript has been judged scientifically suitable for publication and will be formally accepted for publication once it meets all outstanding technical requirements.

Within one week, you’ll receive an e-mail detailing the required amendments. When these have been addressed, you’ll receive a formal acceptance letter and your manuscript will be scheduled for publication.

An invoice will be generated when your article is formally accepted. Please note, if your institution has a publishing partnership with PLOS and your article meets the relevant criteria, all or part of your publication costs will be covered. Please make sure your user information is up-to-date by logging into Editorial Manager at Editorial Manager® and clicking the ‘Update My Information' link at the top of the page. If you have any questions relating to publication charges, please contact our Author Billing department directly at authorbilling@plos.org.

If your institution or institutions have a press office, please notify them about your upcoming paper to help maximize its impact. If they’ll be preparing press materials, please inform our press team as soon as possible -- no later than 48 hours after receiving the formal acceptance. Your manuscript will remain under strict press embargo until 2 pm Eastern Time on the date of publication. For more information, please contact onepress@plos.org.

Kind regards,

Olatunji Matthew Kolawole, Ph.D.

Academic Editor

PLOS ONE

Additional Editor Comments (optional):

Reviewers' comments:

# Editor report: Acceptance letter

**DOI:** 10.1371/journal.pone.0302338.r004

**Authors:** Olatunji Matthew Kolawole

26 Jul 2024

PONE-D-24-12478R1 

PLOS ONE

Dear Dr. Achenbach, 

I'm pleased to inform you that your manuscript has been deemed suitable for publication in PLOS ONE. Congratulations! Your manuscript is now being handed over to our production team.

At this stage, our production department will prepare your paper for publication. This includes ensuring the following:

* All references, tables, and figures are properly cited

* All relevant supporting information is included in the manuscript submission,

* There are no issues that prevent the paper from being properly typeset

If revisions are needed, the production department will contact you directly to resolve them. If no revisions are needed, you will receive an email when the publication date has been set. At this time, we do not offer pre-publication proofs to authors during production of the accepted work. Please keep in mind that we are working through a large volume of accepted articles, so please give us a few weeks to review your paper and let you know the next and final steps. 

Lastly, if your institution or institutions have a press office, please let them know about your upcoming paper now to help maximize its impact. If they'll be preparing press materials, please inform our press team within the next 48 hours. Your manuscript will remain under strict press embargo until 2 pm Eastern Time on the date of publication. For more information, please contact onepress@plos.org.

If we can help with anything else, please email us at customercare@plos.org.

Thank you for submitting your work to PLOS ONE and supporting open access. 

Kind regards, 

PLOS ONE Editorial Office Staff

on behalf of

Dr. Olatunji Matthew Kolawole 

Academic Editor

PLOS ONE
